# Evaluation and treatment of mental health symptoms among unaccompanied migrant children and adolescents in the United States: A systematic review

**DOI:** 10.1002/jcv2.70073

**Published:** 2025-11-20

**Authors:** Natan J. Vega Potler, Sebastian Villegas, Dorice Vieira, Sarah M. Horwitz

**Affiliations:** ^1^ Department of Child and Adolescent Psychiatry New York University Grossman School of Medicine New York New York USA; ^2^ Health Sciences Library New York University Grossman School of Medicine New York New York USA

**Keywords:** immigrants, mental health, psychiatric practice, refugees, trauma

## Abstract

**Background:**

Unaccompanied migrant youths are at elevated risk for exposure to trauma and related mental health challenges, but there is minimal evidence guiding best practices. We conducted a systematic review of quantitative studies that examined mental health evaluation and treatment services for unaccompanied migrant youths resettled in the United States (US).

**Methods:**

In accordance with the PRISMA 2020 statement, a literature search was conducted for empirical studies in nine databases and publicly available search engines. Citation tracking also assisted in identifying additional studies, including from the gray literature. The study protocol was pre‐registered (PROSPERO ID: CRD42023396805). Inclusion criteria required that studies be published between January 1, 2000 and November 11, 2024 and report quantitative results that evaluate mental health status or mental health treatment outcomes for youths (younger than 18 years) who were unaccompanied when they arrived in the US. Descriptive, narrative synthesis was used for analysis of results. Mixed Method Appraisal Tool was used to appraise study quality.

**Results:**

Of 543 studies screened, 13 (*N* = 1494 children)—six cohort studies (retrospective or prospective), five cross‐sectional, one non‐controlled trial, and one program evaluation—met inclusion criteria. Ten evaluated mental health using diverse questionnaires and most focused on trauma‐related symptoms. Three focused on mental health treatment. Findings demonstrated heterogeneous methods of evaluating mental health symptoms and implementing treatment services for unaccompanied migrant youths, although many indicated high rates of reported traumatic exposure. Most reported higher rates of trauma‐related and internalizing symptoms and all treatment studies demonstrated initial promise. Likely due to difficulty in accessing unaccompanied migrant youths, most studies used convenience samples limiting generalizability of findings.

**Conclusions:**

Rigorous studies with comprehensive collection of sociodemographic characteristics are needed to inform the development of evidence‐based services that accurately detect and effectively treat mental health challenges among unaccompanied migrant youths.

## Key points

**What's Known**

- Despite recognition that unaccompanied migrant youths face substantial adversity, there is a dearth of research on how best to support their mental health after resettlement.

**What's New**

- This systematic review highlights high rates of traumatic exposure and heterogeneity with respect to evaluation of mental health and reported sociodemographic characteristics.
- Few interventions have been rigorously evaluated. Some warrant further study, given reductions in mental health symptoms observed.

**What's Relevant**

- This review provides recommendations to enhance clinical care for unaccompanied migrant youths. Efforts to engage youths should consider the high rates of trauma and particular barriers to treatment engagement, including material stressors and language. Expanding rigorous empirical basis for mental health interventions may consider integrating multidisciplinary approaches to attend to challenges experienced by unaccompanied migrant youths.

## INTRODUCTION

In 2020, there were an estimated 36 million child and adolescent migrants globally; nearly 10% of them were living in the United States (US) (United Nations Department of Economic and Social Affairs, 2020). Among this group of migrant youths, unaccompanied minors, *children under 18 years who migrated without a parent or legal guardian*, face unique challenges. The available data suggest that unaccompanied migrant youth (UMY) are at elevated risk of mental health challenges (Menjivar & Perreira, 2019; Sourander, 1998; Vervliet et al., 2014; Zwi & Mares, 2015) and that unaccompanied migration itself is an independent risk factor for the development of psychiatric symptoms (Bean et al., 2007). From January 2021‐December 2023 there were almost 460,000 UMY apprehensions in the US (*U.S. Customs and Border Protection Encounters: FY Southwest Land Border Encounters by Month*, 2024) and an unknown number have entered without being apprehended by government agencies.

Unaccompanied child migration is a global phenomenon, but each destination country varies considerably in the legal, political, medical, and economic policies for receiving youths as well as characteristics of the migrant youths who arrive (e.g., origin countries, ethnicities, languages, journey) (Corona Maioli et al., 2021). In contrast to many countries that have unaccompanied youth from varied regions and countries, most UMY arriving in the US are from Central America (*U.S. Customs and Border Protection Encounters: FY Southwest Land Border Encounters by Month*, 2024). In the US, the Office of Refugee Resettlement (ORR) has been administering programs for UMY since the early 2000s. Youth have access to health insurance while receiving services from ORR, but their access to health insurance after release to sponsors depends upon several characteristics, including state and locality of residence and immigration status (Beier & Fredricks, 2023). Treatment of UMY in the US differs from other countries in the Global North, in part due to the US not ratifying the Union Nations Convention on the Child, which prioritizes the “best interests of the child” (Levinson, 2011). For example, in contrast to most European nations, US does not guarantee a right to legal representation for UMY, which increases risk of deportation (Khayar‐Cámara et al., 2024).

A number of factors contribute to the migration of UMY to the US, including economic deprivation (Farriss, 2022; Salerno Valdez et al., 2015), climate change (López & Giraldo‐Santiago, 2023; Torres & Casey, 2017), community violence (Donato & Lopez, 2024; Salerno Valdez et al., 2015; United Nations High Commissioner on Refugees, 2014), and family reunification (Sotomayor‐Peterson & Montiel‐Carbajal, 2014; Welch, 2024). Such conditions increase the risk of traumatic exposure, which can be compounded by a perilous migration journey (Hernández Hernández, 2019). Additionally, immigrant detention—which many UMY experience—is associated with adverse physical and mental health outcomes (Coulter et al., 2020). After release from detention, the wellbeing of UMY remains in jeopardy. Although many UMY are temporarily released from detention to the care of a sponsor (in some cases a family member), deportation proceedings are initiated and they must navigate complex legal systems (Chen & Gill, 2015; Linton et al., 2018; Schmidt, 2024). Despite often being eligible for legal immigration statuses, many UMY do not receive legal counsel (Beier et al., 2022; Chen & Gill, 2015), and stress related to deportation often persists.

Economic deprivation is a common driver of migration, but many UMY continue to confront financial stressors post‐migration. Pressure to obtain enough money to meet basic needs and/or send to family in their countries of origin results in many working for pay (Hasson et al., 2021; Vidal de Haymes et al., 2018) and can leave UMY vulnerable to exploitation by US employers (Dreier, 2023a, 2023b), including increased exposure to occupational hazards like pesticides or heat stress (McLaurin & Liebman, 2012). This is particularly concerning since UMY make up approximately 10% of the migrant agricultural workforce in the US (McLaurin & Liebman, 2012).

The importance of identifying effective services to diagnose and treat mental health challenges in these vulnerable youths is reinforced by longitudinal evidence that indicates that psychiatric symptomatology is likely a normative response to substantial childhood adversity (Copeland et al., 2023). Even for those UMY who do access mental health services—<10% access any services after release to a sponsor (Frankel et al., 2021)—finding linguistically relevant services can be challenging (Elmore Borbon et al., 2023; Vidal de Haymes et al., 2018). This lack of service access is particularly relevant for youths whose sponsors have insecure immigration status as they often have an understandable distrust of government, including school and/or mental health services (Acuña & Escudero, 2015).

Given that the mental health needs of these youths are considerable, it is important to identify effective mental health screening and treatment services for them. However, there is minimal data guiding best practices for mental health symptom evaluation and intervention in the US. The present systematic review focused on mental health evaluation and treatment services for UMY who have resettled in the US. Specifically, this systematic review sought to identify quantitative studies that examine mental health symptom detection and treatment services for UMY in the US. While the primary focus was on services and symptom detection, the review also included relevant studies that evaluated mental health status outside the context of services.

## METHODS

This report follows The PRISMA 2020 guidelines for reporting systematic reviews (Page et al., 2021) (see Table S1 for PRISMA checklist). The systematic review was prospectively registered in the International Prospective Register of Systematic Reviews (PROSPERO) at https://www.crd.york.ac.uk/PROSPERO/view/CRD42023396805/. A systematic search was conducted in the following databases: PubMed/Medline (Ovid), Embase, the Cochrane Library, the Cumulative Index to Nursing and Allied Health (CINAHL), Global Health (Ovid), APA PsycInfo, Web of Science, Public Affairs Information Service (PAIS) Index, and Sociological Abstracts databases. Publicly available search engines were also used. No date limits were applied. The initial search was run on December 21, 2022, and the final search was run on November 11, 2024. Due to indexing difficulty, a significant amount of effort was given to citation tracking from included references.

### Search strategy

In consultation with the third author, a medical librarian with expertise in systematic reviews, a search strategy of subject headings and keywords was developed based on the following PICO question: *What mental health services have been described for detection and treatment of mental health symptoms among unaccompanied immigrant children resettled in the US?*

- Population: Participants in the included studies were individuals who were children or adolescents (up to 20 years) who entered the US unaccompanied when younger than 18 years.
- Intervention: All included studies described assessment and/or treatment of mental health symptomatology among UMY resettled in the US.
- Outcomes: Mental health status or symptoms, inclusive of symptom change, were the primary outcomes. These were assessed quantitatively using mental health screening questionnaires, psychiatric diagnosis, and symptom scales.

The generic search was constructed (see Table 1) and then adapted for each database (see Table S2). Specific field codes were used in Ovid, PubMed and other databases for precision searching. Citations were uploaded to EndNote for data management.

**TABLE 1:** Generic search strategy.

Concept	Generic search terms
Unaccompanied minors	(unaccompanied OR un‐accompanied OR unattended OR unchaperoned OR unescorted OR abandoned child OR child abandonment OR separated OR alien OR asylum OR family separation OR unaccompanied minor* OR minor*) AND (asylee OR refugees OR refugee OR migrant OR migrants OR immigrants OR immigration OR undocumented OR asylum OR human migration OR refugee camp* OR migration)
Mental health services	(diagnos* OR treatment OR therap* OR assessment OR accessibility OR intervention OR implementation OR screening OR screen OR screened OR evaluation OR programs OR community health centers OR clinics OR services OR programs OR evidence‐based)
Mental health	(mental health OR mental health services OR mental disorders OR stress disorders OR anxiety OR depression OR wellbeing OR “well being” OR post‐traumatic stress OR PTSD OR traumatic stress disorder* OR emotion* OR distress*)
United States	(Appalachian Region OR Alabama OR Georgia OR Kentucky OR Maryland OR New York OR North Carolina OR Ohio OR Pennsylvania OR South Carolina OR Tennessee OR Virginia OR West Virginia OR Great Lakes Region OR Illinois OR Indiana OR Michigan OR Minnesota OR Wisconsin OR Mid‐Atlantic Region OR Delaware OR District of Columbia OR Maryland OR New Jersey OR Midwestern United States OR Iowa OR Kansas OR Kentucky OR Missouri OR Nebraska OR North Dakota OR Oklahoma OR South Dakota OR Wisconsin OR New England OR Connecticut OR Maine OR Massachusetts OR New Hampshire OR Rhode Island OR Vermont OR Idaho OR Montana OR Washington OR Wyoming OR Pacific States OR Alaska OR California OR Hawaii OR Oregon OR Alabama OR Arkansas OR Florida OR Louisiana OR Mississippi OR Arizona OR Colorado OR Nevada OR New Mexico OR Texas OR Utah OR United States OR America* OR “U.S.” OR US)

*Note*: Filters: English, Child: birth–18 years.

### Eligibility criteria

Studies were included if they were any of the following: retrospective and prospective cohort studies, cross‐sectional studies, program evaluations, mixed‐methods, randomized control trials, non‐randomized control trials, and open trials. Studies included were published from January 1, 2000‐November 11, 2024, and included quantitative mental health symptom assessment and/or treatment of UMY. Timeframe was selected based upon US governmental changes at the start of the 21^st^ century that transferred UMY supervision from enforcement agencies to ORR, which is under Health and Human Services. Studies were included if inclusive of participants who migrated to the US as UMY and were children and adolescents (up to 20 years) at the time of evaluation or treatment. Studies that described mental health evaluation within and outside the context of services.

#### Exclusion criteria

Studies were excluded if they did not report quantitative mental health outcomes or if they did not report these for UMY separately (i.e., if non‐UMY were also included in the study sample). They were excluded if they did not focus on unaccompanied youth in the US or the participants were not unaccompanied when they arrived in the US.

### Data extraction and synthesis

Data was exported from EndNote to Covidence, a web‐based systematic review management program. Covidence was used for study screening in two stages, removal of duplicate publications, and data extraction. First, two authors (N. J. V. P. and S. V.) independently reviewed records and excluded titles and abstracts. Second, full text review was independently conducted by two authors (N. J. V. P. and S. V.) to identify the studies that would be included in systematic review for full data extraction. Conflicts were resolved via discussion and, if needed, a third reviewer (D. V.) adjudicated a final decision. Search strategy was tested and refined prior to finalization. Evaluation of inter‐rater reliability of full text review yielded Cohen's *κ* = 0.87. Literature search yielded 906 records, of which 363 were identified as duplicates. Citation tracking identified 12 additional studies. In total, 543 potential records were screened and full text review identified 13 studies that met inclusion criteria.

Two reviewers independently (N. J. V. P. and S. V.) extracted quantitative mental health outcomes, including descriptive statistics on symptoms scales/questionnaires, statistical testing related to intervention, and intervention modality if relevant. Additional extracted data included study focus (evaluation or treatment), study design, sample descriptive statistics (age, gender, origin country, languages spoken), resettled state, mental health domain (e.g., depression), living context, traumatic event exposure, reported suicidality, coping strategies and/or resilience scales. Extraction template can be provided upon request. A descriptive, narrative review was used to synthesize quantitative findings from included studies. Due to methodological and clinical heterogeneity, no meta‐analysis was conducted. Studies published between January 1, 2000, and November 11, 2024, were included.

### Risk of bias assessment

All included studies were assessed formally for risk of bias using the Mixed Methods Appraisal Tool (MMAT) (Hong et al., 2018). A Google form was developed and branched to the appropriate assessment tool based on study design as per the MMAT. Overall quality was systematically calculated by total number of questions with “yes” for each study type, as has been previously reported (Lee et al., 2023). A total of 6–7 questions with “yes” was defined as high quality, 4–5 questions with “yes” were defined as medium quality, and 1–3 questions with “yes” were defined as low quality. Two of three authors (N. J. V. P., S. V., D. V.) independently evaluated all studies using the MMAT. Independent assessments were reviewed, and disagreements were resolved through discussion or consultation with the third author.

## RESULTS

**FIGURE 1:**
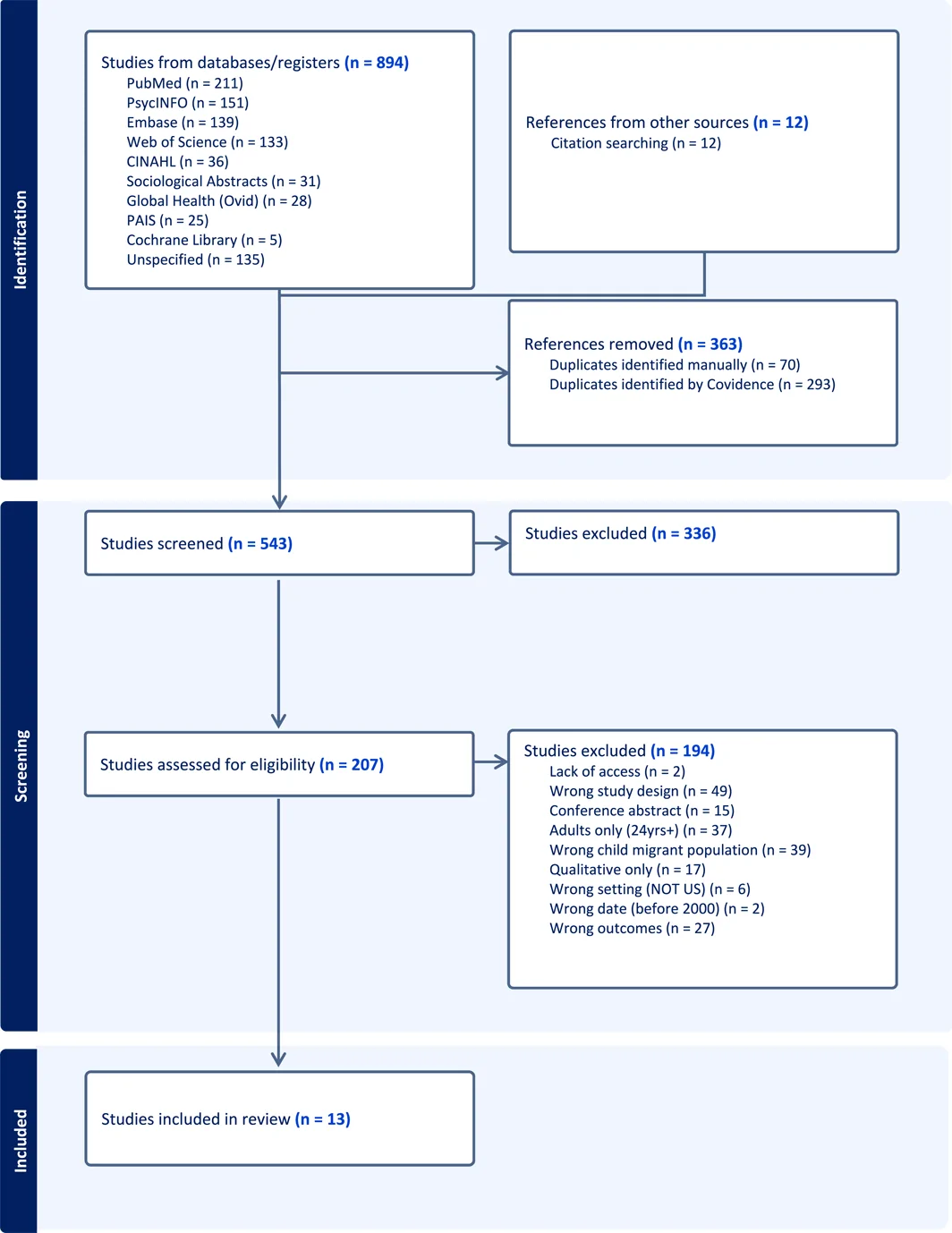
PRISMA flow diagram. CINAHL, Cumulative Index to Nursing and Allied Health Literature; PAIS, Public Affairs Information Service.

This review identified quantitative studies that examine mental health symptom detection and treatment services for UMY in the US. In total, the search strategy and citation tracking identified 906 studies published between January 1, 2000, and November 11, 2024. From these, 13 eligible studies were included (see Figure 1). Eleven studies were published in the peer‐reviewed literature, one was a doctoral dissertation (Baily, 2017), and one was published as a book chapter (Grant‐Knight et al., 2008). Seven studies used primary data, while the remaining six relied upon secondary data from administrative or clinical records. While the stated design in the included study was often consistent with authors' assessments, there were cases in which study design was not explicitly stated or conflicting descriptions were provided. For example, one study was titled as an open‐label trial, but the data was noted to be taken from chart review and was not preplanned. The studies analyzed data collected from states across the country, including New York, Florida, and California. Assessment of included studies revealed variable strengths, limitations, and overall quality. As per MMAT, most studies reported quantitative descriptive analyses. Of all included studies, six were high quality, three were medium quality, and four were low quality (see Table S3). Most limitations pertained to unclear information provided, specifically related to sampling strategy and representativeness, but also for nonresponse bias and analytic methods.

### Participant characteristics and traumatic exposures

Among included studies, the total sample sizes ranged from 6 to 476 UMY (see Table 2). The total sample across studies was 1494, including 1073 males, 382 females, and 39 without reported gender or assigned sex at birth. Two studies (Geltman et al., 2005; Grant‐Knight et al., 2008) reported on the same study sample, resulting in a total of 1190 unique participants. They were from various origin countries. Among the 11 studies that reported origin country, most were from Sudan (44%) and Central America (21%). The two studies that did not report origin countries by number noted that most of the sample were from Central America or Mexico. The mean age of participants was reported in most studies (*n* = 10) and among studies that reported a participant age range, most were older children through young adults (*n* = 5). One study included children as young as 4 years (Patel et al., 2022).

**TABLE 2:** Included study characteristics.

Study ID	Design	Total unaccompanied migrant youths sample (% female)	Resettled state	Region/country of origin	Targeted domain	Traumatic exposure assessed and reported?	Evaluation or treatment	Treatment modality	Summary of quantitative findings
Baily (2017)	Mixed‐methods (cohort study, prospective)	26 (42.3%)	New York	Central America (El Salvador, Guatemala, Honduras) South America (Ecuador)	PTSD Depression Anxiety Externalizing (conduct, oppositionality) Psychiatric diagnoses (via DISC‐IV)	Yes	Evaluation	N/A	Among a sample in deportation proceedings, structured diagnostic interview indicated high rates (54%) of past‐year psychiatric diagnoses with moderate impairment overall and internalizing disorders, and lower rates of externalizing disorders (12%) or any substance use disorder (<5%). Almost all (88%) had at least one traumatic exposure Over three‐quarters of sample were referred for mental health treatment, though 60% did not follow up with services
Bates et al. (2005)	Mixed‐methods (cross‐sectional study)	43 (11.6%)	Michigan	Africa (Sudan)	PTSD	No	Evaluation	N/A	Among a sample in foster care PTSD symptoms were higher than previous reports of children with single traumatic event, and many cited religion as a source of emotional support
Cardoso (2018)	Mixed‐methods (cross‐sectional study)	30 (50%)	Not specified (“large Southwestern city”)	Central America (El Salvador, Guatemala, Honduras) North America (Mexico)	PTSD Depression with suicidality Substance use	Yes	Evaluation	N/A	Among a school‐based sample there were high rates of suspected PTSD (57%), suspected MDD (30%), past‐year suicidal ideation (30%) and low rates of substance use High rate of traumatic exposure (youths reported a mean of eight traumatic events) Certain coping strategies (e.g., avoidant coping and social withdrawal) were associated with increased mental health symptoms and substance use
Descilo et al. (2014)	Cohort study, retrospective	40 (32% of post‐treatment)	Not specified (implied to be Florida)	Not itemized, though “…most were from Mexico or Central America”	PTSD Depression	Yes	Treatment	Trauma Incident reduction	Among sample in detention with average of eight traumatic events, females reported higher PTSD symptoms After 7.5 sessions delivered once or twice weekly, sample reported significant reduction in PTSD and depressive symptoms Relatively low attrition, which was due to solely to external factors (e.g., deportation)
Evans et al. (2018)	Cohort study, retrospective	30 (30%)	Not reported	Central America (El Salvador, Honduras) Africa (Democratic Republic of Congo, Ethiopia, Somalia) Asia (Afghanistan, Myanmar)	Mental health (generally)	No	Evaluation	N/A	Among a sample who had been recently discharged from foster care, most reported positive outlook or feeling happy, but also challenges with sleep, relaxation, and interpersonal relationships and feelings of limited control over life decisions The survey had low response rate and did not include validated mental health symptom scales
Fortuna et al. (2023)	Mixed‐methods (open non‐controlled trial)	6 (16.7%)	Massachusetts	Central America (El Salvador, Guatemala, Honduras) North America (Mexico)	PTSD	Yes (primary trauma)	Treatment	Mindfulness‐based cognitive behavioral therapy	Among a small sample with PTSD, pre/post analyses demonstrated significant reductions in PTSD symptoms after receipt of six or more of a 12‐session therapy There was no attrition among those receiving the therapy
Geltman et al. (2005)	Cross‐sectional study	304 (16%)	Multiple, but not specified	Africa (Sudan)	PTSD Physical and psychosocial wellbeing	Yes	Evaluation	N/A	Among the sample, one‐fifth had suspected PTSD, though many more reported trauma‐related symptoms and more than three‐quarters had possible psychosomatic symptoms High rate of traumatic exposure was reported, including direct and witnessed interpersonal violence (e.g., 76% witnessed close friends or family being killed)
Grant‐Knight et al. (2008)	Cross‐sectional study	476 (15%)	Multiple, but not specified	Africa (Sudan)	PTSD Coping Physical and psychosocial wellbeing	Yes	Evaluation	N/A	Among the sample one‐fifth had probable PTSD and more than three‐quarters had possible psychosomatic symptoms Coping strategies common for almost entire sample were related to religious faith and trying to improve themselves when criticized Less common strategies were more common among those with PTSD than without (e.g., self‐blame, keeping problems to oneself)
Hasson et al. (2021)	Cohort study, retrospective	163 (38.4%)	Nine sites dispersed through Maryland, Massachusetts, New Jersey, Florida, Georgia, North Carolina, South Carolina, Virginia, Texas	Central America (Guatemala, Honduras, El Salvador)	PTSD	No	Evaluation	N/A	Among a sample receiving post‐release services, relatively low suspected rates of PTSD were reported (8%) and varied by country of origin
Orjuela‐Grimm et al. (2022)	Mixed‐methods (cross‐sectional study)	46 (28%)	New York	Central America (El Salvador, Guatemala, Honduras) North America (Mexico) South America (Ecuador)	Psychological wellbeing	Yes (migration motivation)	Evaluation	N/A	Almost half had at least one “potentially problematic” scale on NIH toolbox, which varied by living context Many reported a high level of resilience (59%)
Patel et al. (2022)	Other: “community program evaluation”	138 (55.1%)	Florida	Not specified beyond 79% were from “Central America”	PTSD General psychiatric symptoms	Yes	Treatment	Trauma‐focused Cognitive behavioral therapy	Among a sample of low suspected PTSD diagnosis (14%), pre/post analysis demonstrated significant decreases in PTSD and generalized mental health symptoms after receipt of an average of nine sessions Over 95% reported one or more traumatic events with an average of four events High attrition (42% completed treatment) among those treated due to external (moving) and internal (self‐discontinuation) factors
Schapiro et al. (2018)	Cohort study, retrospective	30 (not reported)	California	Central America (El Salvador, Guatemala, Honduras) North America (Mexico)	Depression Substance use	No	Evaluation	N/A	Among a sample receiving school‐based services with integrated social services, there was a low rate of positive screens for depression and substance use, though over half were referred
Vega Potler et al. (2023)	Cohort study, retrospective	176 (28.4%)	New York	Central America (El Salvador, Guatemala, Honduras, Nicaragua) North America (Dominican Republic, Jamaica, Mexico) South America (Colombia, Ecuador) Africa (Ghana, Sierra Leone, Somalia, The Gambia)	Emotional distress (including symptoms of PTSD, depression, and anxiety)	No	Evaluation	N/A	Among those receiving care in a specialized pediatric clinic with co‐located services, a high proportion (57%) screened positive for emotional distress with increased rate for female compared to male youths Follow up screening remained high (65%), though follow up data was only available for less than half of sample

Abbreviations: DISC‐IV, Diagnostic Interview Schedule for Children Version IV; MDD, major depressive disorder; N/A, not applicable; PTSD, posttraumatic stress disorder.

Sociodemographic characteristics reported across studies were inconsistent. They included whether unaccompanied minors had been apprehended or spent time in detention (*n* = 3), time in the US at evaluation or start of treatment (*n* = 7), whether participants lived with parents, non‐parent family members, or had other arrangements (*n* = 9). Race, ethnicity, and Indigenous identity as well as language were inconsistently reported. Three studies noted that participants spoke an Indigenous language (e.g., Mam) and/or identified as Indigenous, which was particularly relevant for UMY from Guatemala (Fortuna et al., 2023; Orjuela‐Grimm et al., 2022; Schapiro et al., 2018). Among the participants from Sudan, most reported their ethnicity as Dinka. Additionally, just over half of included studies reported how participant race and/or ethnicity was assessed (*n* = 7).

Among the included studies, eight reported some information about participants' trauma exposure. Some studies reported rates of total or type of trauma events, although one described the primary trauma as per DSM‐5 criterion A among a cohort with posttraumatic stress disorder (PTSD) (Fortuna et al., 2023), and one study reported the rate for whom violence was a reason for migration (Orjuela‐Grimm et al., 2022). There was variability in how trauma history was obtained and reported, but high rates of traumatization were reported across studies. Interpersonal violent victimization and threats as well as witnessing violence—including murder or serious injury—were particularly prevalent. Specifically, 61% migrated to escape violence (Orjuela‐Grimm et al., 2022) (*N* = 46), 76% witnessed close family or friends being killed (Geltman et al., 2005), 89% had at least one traumatic experience (Baily, 2017) (*N* = 26), 89% reported witnessing an attack (Grant‐Knight et al., 2008), and 95% reported at least one traumatic event (Patel et al., 2022) (*N* = 129). The mean number of traumatic events experienced by participants was also reported in three studies, ranging from four to eight events, including physical victimization and exposure to natural disaster (Cardoso, 2018; Descilo et al., 2014; Patel et al., 2022).

### Mental health evaluation

Most studies described mental health evaluation of UMY without treatment (*n* = 10). Half of them were cross‐sectional and half were cohort studies. Almost all quantitatively evaluated mental health using questionnaires that probed symptomatology for a specific diagnostic construct, for example, Child Posttraumatic Stress Scale (CPSS) for PTSD, or transdiagnostic psychiatric distress, for example, the Refugee Health Screener. The most commonly evaluated psychiatric domain was PTSD (*n* = 10) followed by depression (*n* = 4). One study evaluated suicidality using a single item from the PHQ‐9 adolescent version and reported a 30% past‐year rate of suicidal ideation (Cardoso, 2018). Despite most studies evaluating PTSD, none reported on dissociative symptoms. Two studies—using the same cohort—reported an extremely high rate of suspected psychosomatic symptoms (Geltman et al., 2005; Grant‐Knight et al., 2008). The studies evaluating substance use reported low rates (Baily, 2017; Cardoso, 2018; Schapiro et al., 2018). Baily (2017), for example, noted that only a single youth (<5% of sample) met criteria for any substance abuse or dependence.

Only one of the included studies used a semi‐structured or structured diagnostic interview to obtain mental health disorder prevalence rates (Baily, 2017). Among a relatively small sample of participants in deportation proceedings, almost 70% met criteria for any past‐year psychiatric diagnosis (symptoms alone), 54% met criteria for a disorder with symptoms and one or more domain of moderate functional impairment, 54% met criteria for any past‐year internalizing disorder, and 12% met criteria for any past‐year externalizing disorder. The most used questionnaire for evaluating mental health symptoms was the CPSS (*n* = 4), which was noted to have good internal reliability (see Table 3). Estimated diagnostic rates varied widely when relying upon questionnaires and symptom scales. For example, the rates of suspected PTSD ranged from 57% on CPSS (Cardoso, 2018), 20% on the Harvard Trauma Questionnaire (Geltman et al., 2005; Grant‐Knight et al., 2008), 14% on Child and Adolescent Trauma Scale (Patel et al., 2022), and 8% on CPSS (Hasson et al., 2020). Additionally, suspected rate of depression was 30% with the PHQ‐9 (Cardoso, 2018) and although no prevalence rate was reported, mean scores for males was above the cutoff on Center for Epidemiological Studies Depression (>/=16) for moderate depressive symptoms and for females above the severe depressive symptom cutoff (>/=24) (Descilo et al., 2014). The use of transdiagnostic mental health scales indicated relatively high rates of concerning symptoms ranging from 40% on the NIH toolbox emotional battery (Orjuela‐Grimm et al., 2022) to 57% on the Refugee Health Screener (Vega Potler et al., 2023). One study did not use a standardized symptom scale; instead it relied upon questions about mood, outlook, sleep and interpersonal relationships (Evans et al., 2018). Most of the sample reported being happy or very happy and having a positive outlook for the future, but many reported difficulties with sleep, relaxation, or interpersonal relationships (e.g., fear of abandonment). Studies variably examined differences across sociodemographic characteristics or living context. Symptom differences were reported by assigned sex at birth (Vega Potler et al., 2023), post‐resettlement living situation (Orjuela‐Grimm et al., 2022), and origin country (Hasson et al., 2020).

**TABLE 3:** Mental health scales and screeners' mental health domain and internal reliability.

Mental health domain	Mental health scale/screener	Cronbach alpha
Posttraumatic stress disorder	Child Posttraumatic Stress Scale (CPSS)	.85; .87; .93; .95
	Harvard Trauma Questionnaire (HTQ)	NR
	Child and Adolescent Trauma Scale (CATS)	.90 (self)
		.91 (caregiver)
	Posttraumatic Checklist (PCL)	NR
	Posttraumatic Cognitions Inventory (PTCI)	.95
	Reaction of Adolescents to Traumatic Stress (RATS) Total Scale	.66
Depression	Center for Epidemiological Study Depression (CES‐D)	NR
	Patient Health Questionnaire‐9 item (PHQ‐9)	.86
General psychiatric distress	Refugee Health Screener‐15 item (RHS‐15)	NR
	NIH toolbox‐emotional battery	NR
	Hopkins Symptom Checklist‐37a (HSCL‐37a) Total Scale	.85
	Child Behavior Checklist (CBCL) Total Scale	.96
	Strengths and Difficulties Questionnaire (SDQ) Total Difficulties	.80 (self)
		.87 (caregiver)

Abbreviation: NR, not reported.

Two studies included follow‐up information about mental health referral and/or services follow‐up (Baily, 2017; Evans et al., 2018). These indicated high rates of mental health referral (>75%), but less than half had confirmed follow up (40%). Additionally, after discharge from foster care, over one‐quarter received therapy or counseling, <7% received psychotropics, and none were psychiatrically hospitalized or attended substance rehabilitation.

### Coping strategies or resilience

Of the included studies, four evaluated coping strategies or resilience quantitatively with heterogeneity in assessment method (Bates et al., 2005; Cardoso, 2018; Grant‐Knight et al., 2008; Orjuela‐Grimm et al., 2022). One study noted many participants (59%) reported high resilience (Orjuela‐Grimm et al., 2022). Another highlighted that almost the entire sample cited religious faith as an important coping strategy (Grant‐Knight et al., 2008). A study examining the relationship between coping strategies and mental health outcomes, observed that maladaptive strategies (specifically social withdrawal and avoidant coping) were associated with increased psychiatric symptoms, while adaptive coping was associated with decreased substance use (Cardoso, 2018).

### Mental health treatment

The review included three studies on interventions for mental health symptoms among UMY (Descilo et al., 2014; Fortuna et al., 2023; Patel et al., 2022). They were Trauma‐focused CBT (TF‐CBT), Traumatic Incident Reduction (TIR), and Mindfulness Based Cognitive Behavioral Therapy (MB‐CBT) for PTSD and all individual treatments. TF‐CBT and TIR were delivered to unaccompanied minors irrespective of diagnosis, while MB‐CBT was delivered to UMY with PTSD. The studies differed in the symptom scales used and none were randomized controlled trials. Two studies used clinical chart reviews of retrospective cohorts, while one was an open‐label, non‐controlled trial. All studies performed pre/post analyses and reported statistically significant decreases in PTSD (*n* = 3), depressive (*n* = 1) or generalized mental health symptoms (*n* = 1). Two studies also included modifications or adaptations to modalities, for example, education about the foster care or immigration systems was incorporated into TF‐CBT.

TF‐CBT was delivered to participants—living in shelters or with sponsors—who had a low suspected prevalence rate of PTSD pre‐treatment compared to other included studies (Patel et al., 2022). Among the sample who completed treatment, there was a significant decrease in trauma symptoms with a large effect size (−1.03; 95% CI: −1.41 to −0.64). In contrast to the retrospective cohort design, randomized controlled trials of TF‐CBT internationally have most commonly reported moderate effect sizes for improvement in PTSD symptoms (de Arellano et al., 2014; John‐Baptiste Bastien et al., 2020), although higher effect sizes have been observed with less active comparison groups (de Arellano et al., 2014). In addition to the lack of a control group in included studies, there was high rate of attrition for follow‐up assessment (42% completed the treatment). External factors impacted the follow‐up assessments (i.e., a youth leaving the shelter or moving outside the catchment area) in many cases; however, almost 40% of youths who did not complete treatment self‐discontinued and no motivations regarding this were reported. An average of nine sessions were completed, with a wide range from 1 to 48. Of note, less than half of those who completed treatment finished a trauma narrative.

MB‐CBT was developed as a method to incorporate cognitive and mindfulness‐based approaches for adolescents with comorbid PTSD and substance use (Fortuna et al., 2018). It has shown potential clinical effectiveness (reduction in PTSD symptoms, depressive symptoms, and cannabis use) in a small, open label, non‐controlled trial with medium effect sizes (Fortuna et al., 2018). The included study was a mixed‐method analysis of UMY from the initial trial (*N* = 6) with a comparison subsample of US‐born English dominant youths (*N* = 14). Using CPSS and Posttraumatic Cognitions Inventory, a statistically significant decrease in symptoms was observed with medium to large effect sizes after receiving at least six of a 12‐session therapy. The effect sizes for the UMY were much higher for overall PTSD symptoms reduction compared to the US‐born subsample.

TIR was the only intervention delivered exclusively to UMY in a detention center (Descilo et al., 2014). The included study was the first that examined TIR in children and adolescents, and the modality differs from others in its method of exposure and the absence of coping skills. They reported statistically significant reductions in PTSD symptoms (regardless of initial PTSD diagnosis) and depressive symptoms. Female participants had higher pre‐treatment scores and a larger symptom reduction compared to male participants. Treatment discontinuation was reportedly due to external factors (i.e., deportation or being sent to a relative). The average number of sessions was 7.5 and occurred one to two times weekly.

## DISCUSSION

This systematic review focused on mental health evaluation and treatment for UMY resettled in the US. Search of nine databases and the gray literature yielded 13 studies with quantitative mental health outcomes. Most of these evaluated mental health, while three focused on a treatment modality. This review highlights the substantial heterogeneity in reported participant sociodemographic characteristics.

Racial and ethnic reporting varied across studies. Indigenous heritage or language was noted among the sample in less than one‐quarter of studies, despite its relevance for migrants from Latin America, due to the colonial history, genocide, and discrimination experienced by Indigenous peoples (e.g., in Guatemala) (Millender & Lowe, 2017). This is pertinent to clinical practice and research, since some persons may need Indigenous language interpreters (Gentry, 2015) and study designs risk demographic erasure based on US‐centric racial categorization (Barillas Chón, 2019). Other characteristics (e.g., duration of time in the US, living context) were similarly inconsistent despite the relevance for mental health evaluation and treatment. For example, one paper suggested that clinical observations of a “honeymoon” period of adjustment (Polcher & Calloway, 2016) among UMY might impact reported psychiatric symptomatology severity (Schapiro et al., 2018). Another observed that follow‐up screening for emotional distress revealed half of youths who initially screened negative had emergent (i.e., new) positive screening results (Vega Potler et al., 2023). Some characteristics, for example, living context, also have clinical implications; they determine the extent to which a caring attachment figure can be engaged in mental health treatment. The variability of sociodemographic characteristics obtained or reported across studies may also be due in part to the high numbers of studies that used secondary data from administrative or clinical databases. Thus, future primary data collection should comprehensively obtain such information to investigate putative risk, protective, and—importantly—modifiable factors for severe psychiatric distress.

Across studies assessing traumatic events, high rates of traumatization were reported. Consistent with this, the psychiatric domain most frequently evaluated was PTSD followed by depression. Different symptom scales were used to evaluate mental health; the CPSS was used most often and demonstrated good internal reliability across four studies (Bates et al., 2005; Cardoso, 2018; Hasson et al., 2020). Despite many studies focusing on traumatic symptoms, none included a measure that detected dissociative symptoms, despite longitudinal observations that dissociative symptoms are associated with self‐injury (Tanaka et al., 2024). Of particular concern, a high rate of suicidal ideation was reported in the single study in which it was evaluated (Cardoso, 2018). Similarly, suspected psychosomatic symptoms were reported among over three‐quarters of participants. Most studies did not provide further information about treatment after evaluation or screening in the context of services. Thus, further investigations to guide evidence‐based mental health screening and follow up treatment practices are warranted.

Among the included treatment studies, none were randomized or had a control group. They either used secondary data analysis with pre/post analyses or used an open‐label, non‐controlled trial design. The included studies provided nascent evidence for trauma‐focused interventions, though their findings were limited by study design, sample size, and attrition. The findings were notable due to the scarcity of studies focusing on treatment services generally as well as “gold‐standard” interventions for UMY. One potential contributory factor to the limited implementation of evidence‐based services for UMY may be related to challenges in following up with weekly therapy using manualized interventions when children are fighting to meet basic needs (Muñiz de la Peña et al., 2019). Because of the importance of considering the material conditions and practices of communities in implementation science (Sanchez et al., 2023), it was notable that no treatment studies integrated multidisciplinary services with mental health care, though such models exist (Beier et al., 2022; Linton et al., 2018). For example, having an immigration attorney is essential to obtaining lawful immigration status in the US (Grace & Roth, 2015). In addition to reporting fidelity to evidence‐based modalities, future studies should—as reported in two treatment studies—note modifications or adaptations. Identification of protective factors (e.g., living with caring adult family (Sotomayor‐Peterson & Montiel‐Carbajal, 2014)) and building on sources of resilience will be important for designing effective services. Lastly, the interventions in included studies were all individual‐focused modalities, although data from UMY internationally suggests that group interventions are particularly promising (Hutchinson et al., 2022).

### Systematic review limitations

This systematic review has limitations. A substantial proportion of the current knowledge of UMY in the US is informed by research on the “Lost Boys” of Sudan, who were resettled in the early 2000s after spending time in refugee camps in Ethiopia or Kenya (*n* = 3; *N* = 823 across the studies). Although insights from this research are important, the population differs from most UMY in the US, who are mostly from Central America. Many of the evaluation studies were based on convenience samples or secondary analyses of administrative or clinical data, which could bias the findings and limit generalizability. Because UMY in the US are a “hard‐to‐reach” or “hidden” population such sampling biases are expected. However, reliability of prevalence rates is unclear and only one included study used a semi‐structured evaluation for psychiatric diagnosis. Community‐based participatory methods may be particularly helpful for more robust recruitment. The focus of the present systematic review was about UMY in the US, so methods of mental health evaluation or treatment in other settings, for example, during migration, may not have been identified. The present review did not examine the impact of evolving US immigration policies on mental health services, but such investigations are warranted. Although the use of four elements in the search strategy can have limitations for complex topics, multiple efforts were undertaken to mitigate these, including the involvement of an expert medical librarian from initial stages of the review who optimized search strategy iteratively, searches of the gray literature, and citation tracking.

### Implications for future research and clinical services

The findings from this systematic review yield several recommendations for empirical research and clinical care of UMY. Primary data collection studies should optimize collection of sociodemographic characteristics by ensuring that the characteristics obtained are relevant to the target UMY population and avoiding overreliance on host country's racial categorizations. For example, US‐based studies may use racial and ethnic categories that lead to the demographic erasure of Indigenous migrants from Latin America. Studies should optimize collection of post‐resettlement factors that may impact mental health, for example, living context, legal representation, work or school involvement. Additionally, prospective, longitudinal studies could examine whether a “honeymoon period”—those in which youths have emergent or worsening of mental health symptoms the longer they are from arrival—impacts mental health screening and treatment response.

Studies highlight substantial adversity faced by UMY. Trauma‐related sequelae and internalizing rather than externalizing symptoms are more frequently reported in the literature. Future research should include evidence‐based, validated measures of potentially relevant behaviors and symptoms, including suicidality, self‐injury, dissociation, and psychosomatic symptoms.

Mental health services for UMY should consider universal trauma screening and trauma‐informed care given high rates of reported trauma. Factors that impact the disclosure of traumatic experiences by UMY also warrant further investigation. Information on psychometrics for screeners and scales as well as adaptations or modifications of evidence‐based treatments will be important for optimizing symptom evaluation and interventions. Lastly, clinical approaches should leverage the resilience of youths while also attending to factors that may impact engagement, whether political, linguistic, cultural, or material. For example, interdisciplinary services relevant to youths' particular needs could be integrated with mental health service delivery (e.g., medical‐legal partnerships).

## CONCLUSIONS

The findings highlight heterogeneous methods of evaluating mental health symptoms among UMY and the limited data on mental health treatment services. Additionally, they indicate that further research on methods of screening and treating psychiatric symptomatology among UMY is warranted. Importantly, even these limited data show high rates of mental health challenges facing UMY and demonstrate that establishing an evidence base for clinical services for unaccompanied migrant youths is critical.

## AUTHOR CONTRIBUTIONS

**Natan J. Vega Potler**: Conceptualization; formal analysis; funding acquisition; methodology; project administration; writing—original draft; writing—review and editing. **Sebastian Villegas**: Data curation; formal analysis; project administration; writing—review and editing. **Dorice Vieira**: Conceptualization; data curation; investigation; methodology; software; writing—review and editing. **Sarah M. Horwitz**: Conceptualization; methodology; supervision; writing—review and editing.

## CONFLICT OF INTEREST STATEMENT

N. J. V. P. reported receipt of funding from the NYU Center for the Study of Asian American Health under the NIH/NIMHD grant award U54MD000538 outside of the submitted work. S. M. H. receives royalties from American Psychiatric Association Publishing. The remaining authors have declared that they have no competing or potential conflicts of interest.

## ETHICAL CONSIDERATIONS

No ethics approval or patient consent were required as no new data were collected for this systematic review.

## PRE‐REGISTRATION DETAILS

The systematic review was prospectively registered on 2/18/23, PROSPERO ID: CRD42023396805, in the International Prospective Register of Systematic Reviews (PROSPERO) at https://www.crd.york.ac.uk/PROSPERO/view/CRD42023396805/.

## Supporting information

Tables S1–S3

## Data Availability

The data that support these findings are openly available in public domain.
