# Supplementary material for: Evaluation and treatment of mental health symptoms among unaccompanied migrant children and adolescents in the United States: A systematic review
Source: JCPP Adv. 2025 Nov 20;6(3):e70073. doi: 10.1002/jcv2.70073 (PMC13339222; doi:10.1002/jcv2.70073)
Supplement: Supplementary file 1 [file JCV2-6-e70073-s001.docx]

**Supporting Information**

**TableS1. PRISMA Checklist**

| **Section and Topic** | **Item #** | **Checklist item** | **Location where item is reported** |
| --- | --- | --- | --- |
| **TITLE** | | |  |
| Title | 1 | Identify the report as a systematic review. | Title |
| **ABSTRACT** | | |  |
| Abstract | 2 | See the PRISMA 2020 for Abstracts checklist. | Abstract |
| **INTRODUCTION** | | |  |
| Rationale | 3 | Describe the rationale for the review in the context of existing knowledge. | Introduction |
| Objectives | 4 | Provide an explicit statement of the objective(s) or question(s) the review addresses. | Introduction |
| **METHODS** | | |  |
| Eligibility criteria | 5 | Specify the inclusion and exclusion criteria for the review and how studies were grouped for the syntheses. | Methods |
| Information sources | 6 | Specify all databases, registers, websites, organisations, reference lists and other sources searched or consulted to identify studies. Specify the date when each source was last searched or consulted. | Methods |
| Search strategy | 7 | Present the full search strategies for all databases, registers and websites, including any filters and limits used. | Methods/Table 1 (generic search strategy); Supplementary File (search strategies by database) |
| Selection process | 8 | Specify the methods used to decide whether a study met the inclusion criteria of the review, including how many reviewers screened each record and each report retrieved, whether they worked independently, and if applicable, details of automation tools used in the process. | Methods |
| Data collection process | 9 | Specify the methods used to collect data from reports, including how many reviewers collected data from each report, whether they worked independently, any processes for obtaining or confirming data from study investigators, and if applicable, details of automation tools used in the process. | Methods |
| Data items | 10a | List and define all outcomes for which data were sought. Specify whether all results that were compatible with each outcome domain in each study were sought (e.g. for all measures, time points, analyses), and if not, the methods used to decide which results to collect. | Methods |
|  | 10b | List and define all other variables for which data were sought (e.g. participant and intervention characteristics, funding sources). Describe any assumptions made about any missing or unclear information. | Methods |
| Study risk of bias assessment | 11 | Specify the methods used to assess risk of bias in the included studies, including details of the tool(s) used, how many reviewers assessed each study and whether they worked independently, and if applicable, details of automation tools used in the process. | Methods |
| Effect measures | 12 | Specify for each outcome the effect measure(s) (e.g. risk ratio, mean difference) used in the synthesis or presentation of results. | Methods; Table 2 |
| Synthesis methods | 13a | Describe the processes used to decide which studies were eligible for each synthesis (e.g. tabulating the study intervention characteristics and comparing against the planned groups for each synthesis (item #5)). | Methods |
|  | 13b | Describe any methods required to prepare the data for presentation or synthesis, such as handling of missing summary statistics, or data conversions. | Methods |
|  | 13c | Describe any methods used to tabulate or visually display results of individual studies and syntheses. | Methods |
|  | 13d | Describe any methods used to synthesize results and provide a rationale for the choice(s). If meta-analysis was performed, describe the model(s), method(s) to identify the presence and extent of statistical heterogeneity, and software package(s) used. | Methods |
|  | 13e | Describe any methods used to explore possible causes of heterogeneity among study results (e.g. subgroup analysis, meta-regression). | N/A |
|  | 13f | Describe any sensitivity analyses conducted to assess robustness of the synthesized results. | N/A |
| Reporting bias assessment | 14 | Describe any methods used to assess risk of bias due to missing results in a synthesis (arising from reporting biases). | N/A |
| Certainty assessment | 15 | Describe any methods used to assess certainty (or confidence) in the body of evidence for an outcome. | N/A |
| **RESULTS** | | |  |
| Study selection | 16a | Describe the results of the search and selection process, from the number of records identified in the search to the number of studies included in the review, ideally using a flow diagram. | Results; Figure 1 |
|  | 16b | Cite studies that might appear to meet the inclusion criteria, but which were excluded, and explain why they were excluded. | N/A |
| Study characteristics | 17 | Cite each included study and present its characteristics. | Results; Table 2 |
| Risk of bias in studies | 18 | Present assessments of risk of bias for each included study. | Results; TableS3 |
| Results of individual studies | 19 | For all outcomes, present, for each study: (a) summary statistics for each group (where appropriate) and (b) an effect estimate and its precision (e.g. confidence/credible interval), ideally using structured tables or plots. | Results; Table 2 |
| Results of syntheses | 20a | For each synthesis, briefly summarise the characteristics and risk of bias among contributing studies. | Results; TableS3 |
|  | 20b | Present results of all statistical syntheses conducted. If meta-analysis was done, present for each the summary estimate and its precision (e.g. confidence/credible interval) and measures of statistical heterogeneity. If comparing groups, describe the direction of the effect. | N/A |
|  | 20c | Present results of all investigations of possible causes of heterogeneity among study results. | N/A |
|  | 20d | Present results of all sensitivity analyses conducted to assess the robustness of the synthesized results. | N/A |
| Reporting biases | 21 | Present assessments of risk of bias due to missing results (arising from reporting biases) for each synthesis assessed. | N/A |
| Certainty of evidence | 22 | Present assessments of certainty (or confidence) in the body of evidence for each outcome assessed. | N/A |
| **DISCUSSION** | | |  |
| Discussion | 23a | Provide a general interpretation of the results in the context of other evidence. | Discussion |
|  | 23b | Discuss any limitations of the evidence included in the review. | Discussion |
|  | 23c | Discuss any limitations of the review processes used. | Discussion |
|  | 23d | Discuss implications of the results for practice, policy, and future research. | Discussion |
| **OTHER INFORMATION** | | |  |
| Registration and protocol | 24a | Provide registration information for the review, including register name and registration number, or state that the review was not registered. | Methods |
|  | 24b | Indicate where the review protocol can be accessed, or state that a protocol was not prepared. | Methods |
|  | 24c | Describe and explain any amendments to information provided at registration or in the protocol. | N/A |
| Support | 25 | Describe sources of financial or non-financial support for the review, and the role of the funders or sponsors in the review. | Acknowledgements |
| Competing interests | 26 | Declare any competing interests of review authors. | Conflicts of Interest |
| Availability of data, code and other materials | 27 | Report which of the following are publicly available and where they can be found: template data collection forms; data extracted from included studies; data used for all analyses; analytic code; any other materials used in the review. | Methods |

*From:*  Page MJ, McKenzie JE, Bossuyt PM, Boutron I, Hoffmann TC, Mulrow CD, et al. The PRISMA 2020 statement: an updated guideline for reporting systematic reviews. BMJ 2021;372:n71. doi: 10.1136/bmj.n71. This work is licensed under CC BY 4.0. To view a copy of this license, visit <https://creativecommons.org/licenses/by/4.0/>

**TableS2. Search Strategies by Database**

| **PubMed** |  |  |
| --- | --- | --- |
| [TW] searches the title, abstract, MESH, and author keywords fields | |  |
| **History and Search Details** | |  |
| **Search** | **Query** | **Results** |
| #2 | Search: ((Appalachian Region [TW] OR Alabama [TW] OR Georgia [TW] OR Kentucky [TW] OR Maryland [TW] OR New York [TW] OR North Carolina [TW] OR Ohio [TW] OR Pennsylvania [TW] OR South Carolina [TW] OR Tennessee [TW] OR Virginia [TW] OR West Virginia [TW] OR Great Lakes Region [TW] OR Illinois [TW] OR Indiana [TW] OR Michigan [TW] OR Minnesota [TW] OR Wisconsin [TW] OR Mid-Atlantic Region [TW] OR Delaware [TW] OR District of Columbia [TW] OR Maryland [TW] OR New Jersey [TW] OR Midwestern United States [TW] OR Iowa [TW] OR Kansas [TW] OR Kentucky [TW] OR Missouri [TW] OR Nebraska [TW] OR North Dakota [TW] OR Oklahoma [TW] OR South Dakota [TW] OR Wisconsin [TW] OR New England [TW] OR Connecticut [TW] OR Maine [TW] OR Massachusetts [TW] OR New Hampshire [TW] OR Rhode Island [TW] OR Vermont [TW] OR Idaho [TW] OR Montana [TW] OR Washington [TW] OR Wyoming [TW] OR Pacific States [TW] OR Alaska [TW] OR California [TW] OR Hawaii [TW] OR [tw] Oregon [TW] OR Alabama [TW] OR Arkansas [TW] OR Florida [tw] OR Louisiana [TW] OR Mississippi [TW] OR Arizona [TW] OR Colorado [tw] OR Nevada [tw] OR New Mexico [tw] OR Texas [tw] OR Utah [tw]) OR ((United States [tw] OR United States [MESH] OR America* [tw] OR "U.S." [tw] OR US [tw])) AND (english[Filter])) AND ((diagnos* OR treatment OR therap* OR assessment OR accessibility OR intervention OR implementation OR screening OR screen OR screened OR evaluation OR programs OR community health centers OR clinics OR services OR programs OR evidence-based) AND (mental health OR mental health services OR mental disorders OR stress disorders OR anxiety OR depression OR wellbeing OR "well being" OR post-traumatic stress OR PTSD OR traumatic stress disorder* OR emotion* OR distress*) AND (unaccompanied [tw] OR un-accompanied [tw] OR unattended [tw] OR unchaperoned [tw] OR unescorted [tw] OR abandoned child [tw] OR child abandonment OR separated [tw] OR alien [tw] OR asylum [tw] OR family separation [tw]) AND (refugees OR refugee OR migrant OR migrants OR immigrants OR immigration OR undocumented OR asylum OR human migration OR refugee camp* OR migration) AND (english[Filter])) Sort by: First Author | [380](https://pubmed.ncbi.nlm.nih.gov/?term=longquery4a9e337c9e0449806f12&sort=fauth&size=200) |
| #1 | Search: ((Appalachian Region [TW] OR Alabama [TW] OR Georgia [TW] OR Kentucky [TW] OR Maryland [TW] OR New York [TW] OR North Carolina [TW] OR Ohio [TW] OR Pennsylvania [TW] OR South Carolina [TW] OR Tennessee [TW] OR Virginia [TW] OR West Virginia [TW] OR Great Lakes Region [TW] OR Illinois [TW] OR Indiana [TW] OR Michigan [TW] OR Minnesota [TW] OR Wisconsin [TW] OR Mid-Atlantic Region [TW] OR Delaware [TW] OR District of Columbia [TW] OR Maryland [TW] OR New Jersey [TW] OR Midwestern United States [TW] OR Iowa [TW] OR Kansas [TW] OR Kentucky [TW] OR Missouri [TW] OR Nebraska [TW] OR North Dakota [TW] OR Oklahoma [TW] OR South Dakota [TW] OR Wisconsin [TW] OR New England [TW] OR Connecticut [TW] OR Maine [TW] OR Massachusetts [TW] OR New Hampshire [TW] OR Rhode Island [TW] OR Vermont [TW] OR Idaho [TW] OR Montana [TW] OR Washington [TW] OR Wyoming [TW] OR Pacific States [TW] OR Alaska [TW] OR California [TW] OR Hawaii [TW] OR [tw] Oregon [TW] OR Alabama [TW] OR Arkansas [TW] OR Florida [tw] OR Louisiana [TW] OR Mississippi [TW] OR Arizona [TW] OR Colorado [tw] OR Nevada [tw] OR New Mexico [tw] OR Texas [tw] OR Utah [tw]) OR ((United States [tw] OR United States [MESH] OR America* [tw] OR "U.S." [tw] OR US [tw])) AND (**(diagnos* [tw] OR treatment [tw] OR therap* [tw] OR assessment [tw] OR accessibility [tw] OR intervention [tw] OR implementation [tw] OR screening [tw] OR screen [tw] OR screened [tw] OR evaluation [tw] OR programs [tw] OR community health centers [tw] OR clinics [tw] OR services [tw] OR programs [tw] OR evidence-based [tw])** AND (mental health OR mental health services OR mental disorders OR stress disorders OR anxiety OR depression OR wellbeing OR "well being" OR post-traumatic stress OR PTSD OR traumatic stress disorder* OR emotion* OR distress*) AND (unaccompanied [tw] OR un-accompanied [tw] OR unattended [tw] OR unchaperoned [tw] OR unescorted [tw] OR abandoned child [tw] OR child abandonment OR separated [tw] OR alien [tw] OR asylum [tw] OR family separation [tw]) AND (refugees OR refugee OR migrant OR migrants OR immigrants OR immigration OR undocumented OR asylum OR human migration OR refugee camp* OR migration) AND (english[Filter])) Sort by: First Author | [300](https://pubmed.ncbi.nlm.nih.gov/?term=longquery9faab47d942d9906dca9&sort=fauth&sort_order=asc&size=200) |
|  |  |  |
| **Web of Science, PAIS, Sociological Abstracts, CINAHL   (This is the generic search strategy)** | (mental health OR mental health services OR mental disorders OR stress disorders OR anxiety OR depression OR wellbeing OR "well being" OR post-traumatic stress OR PTSD OR traumatic stress disorder* OR emotion* OR distress*) AND (unaccompanied OR un-accompanied OR unattended OR unchaperoned OR unescorted OR abandoned child OR child abandonment OR separated OR alien OR asylum seeking) AND (asylee OR refugees OR refugee OR migrant OR migrants OR immigrants OR immigration OR undocumented OR asylum OR human migration OR refugee camp* OR migration) (Abstract) or (mental health OR mental health services OR mental disorders OR stress disorders OR anxiety OR depression OR wellbeing OR "well being" OR post-traumatic stress OR PTSD OR traumatic stress disorder* OR emotion* OR distress*) AND (unaccompanied OR un-accompanied OR unattended OR unchaperoned OR unescorted OR abandoned child OR child abandonment OR separated OR alien OR asylum seeking) AND (asylee OR refugees OR refugee OR migrant OR migrants OR immigrants OR immigration OR undocumented OR asylum OR human migration OR refugee camp* OR migration) (Title) and Child* Or Youth Or Adolescen* OR Young Adult OR Teens OR Teenager* (Search within all fields) and United States OR Usa OR "U.s.a." OR "U.s." (Search within all fields) |  |
|  |  |  |
| **Ovid Databases: Embase, APA PsycInfo, Cochrane and Global Health** | (Appalachian Region.ti,ab,hw. OR Alabama.ti,ab,hw. OR Georgia.ti,ab,hw. OR Kentucky.ti,ab,hw. OR Maryland.ti,ab,hw. OR New York.ti,ab,hw. OR North Carolina.ti,ab,hw. OR Ohio.ti,ab,hw. OR Pennsylvania.ti,ab,hw. OR South Carolina.ti,ab,hw. OR Tennessee.ti,ab,hw. OR Virginia.ti,ab,hw. OR West Virginia.ti,ab,hw. OR Great Lakes Region.ti,ab,hw. OR Illinois.ti,ab,hw. OR Indiana.ti,ab,hw. OR Michigan.ti,ab,hw. OR Minnesota.ti,ab,hw. OR Wisconsin.ti,ab,hw. OR Mid-Atlantic Region.ti,ab,hw. OR Delaware.ti,ab,hw. OR District of Columbia.ti,ab,hw. OR Maryland.ti,ab,hw. OR New Jersey.ti,ab,hw. OR Midwestern United States.ti,ab,hw. OR Iowa.ti,ab,hw. OR Kansas.ti,ab,hw. OR Kentucky.ti,ab,hw. OR Missouri.ti,ab,hw. OR Nebraska.ti,ab,hw. OR North Dakota.ti,ab,hw. OR Oklahoma.ti,ab,hw. OR South Dakota.ti,ab,hw. OR New England.ti,ab,hw. OR Connecticut.ti,ab,hw. OR Maine.ti,ab,hw. OR Massachusetts.ti,ab,hw. OR New Hampshire.ti,ab,hw. OR Rhode Island.ti,ab,hw. OR Vermont.ti,ab,hw. OR Idaho.ti,ab,hw. OR Montana.ti,ab,hw. OR Washington.ti,ab,hw. OR Wyoming.ti,ab,hw. OR Pacific States.ti,ab,hw. OR Alaska.ti,ab,hw. OR California.ti,ab,hw. OR Hawaii.ti,ab,hw. OR Oregon.ti,ab,hw. OR Alabama.ti,ab,hw. OR Arkansas.ti,ab,hw. OR Florida.ti,ab,hw. OR Louisiana.ti,ab,hw. OR Mississippi.ti,ab,hw. OR Arizona.ti,ab,hw. OR Colorado.ti,ab,hw. OR Nevada.ti,ab,hw. OR New Mexico.ti,ab,hw. OR Texas.ti,ab,hw. OR Utah.ti,ab,hw. OR United States.ti,ab,hw. OR America*.ti,ab,hw. OR "U.S.".ti,ab,hw. OR US.ti,ab,hw.) AND (diagnos* OR treatment OR therap* OR assessment OR accessibility OR intervention OR implementation OR screening OR screen OR screened OR evaluation OR programs OR community health centers OR clinics OR services OR programs OR evidence-based) AND (mental health OR mental health services OR mental disorders OR stress disorders OR anxiety OR depression OR wellbeing OR "well being" OR post-traumatic stress OR PTSD OR traumatic stress disorder* OR emotion* OR distress*) AND **(unaccompanied.ti,ab,hw. OR unattended.ti,ab,hw. OR unchaperoned.ti,ab,hw. OR unescorted.ti,ab,hw. OR abandoned child.ti,ab,hw. OR child abandonment.ti,ab,hw. OR separated.ti,ab,hw. OR alien.ti,ab,hw. OR asylum.ti,ab,hw. OR family separation.ti,ab,hw.)** AND (refugees OR refugee OR migrant OR migrants OR immigrants OR immigration OR undocumented OR asylum OR human migration OR refugee camp* OR migration) |  |

| **MIXED METHODS STUDIES** | | | | | | | | | |
| --- | --- | --- | --- | --- | --- | --- | --- | --- | --- |
| **Study ID** | **Clear research questions?** | **Collected data allow the research questions to be addressed?** | **Adequate rationale for using a mixed methods design to address the research question?** | **Different components of the study are effectively integrated to answer the research question?** | **Outputs of the integration of qualitative and quantitative components are adequately interpreted?** | **Divergences and inconsistencies between quantitative and qualitative results are adequately addressed?** | **Different components of the study adhere to the quality criteria of each tradition of the methods involved?** | **Quality** | **Comments** |
| Baily 2017 | Yes | Yes | Yes | Yes | Yes | Yes | Yes | High | Clear rationale, integration of findings, discussion of divergences. |
| Bates et al 2005 | Yes | Yes | No | No | No | Yes | No | Low | Quantitative and qualitative findings were not clearly integrated. Quantitative findings were minimally discussed. |
| Cardoso 2018 | Yes | Yes | Yes | Yes | Yes | Yes | Yes | High | Clear rationale, integration of findings, discussion of divergences. |
| Fortuna et al 2023 | Yes | Yes | Yes | Yes | Yes | Yes | Yes | High | Sample size limitations |
| Orjuela-Grimm et al 2022 | Yes | Yes | Yes | Yes | Yes | Yes | Yes | High | Clear rationale, integration of findings, discussion of divergences. Limitations in sample and generalizability. |
|  |  |  |  |  |  |  |  |  |  |
| **QUANTITATIVE DESCRIPTIVE STUDIES** | | | | | | | | | |
| **Study ID** | **Clear research questions?** | **Collected data allow the research questions to be addressed?** | **Sampling strategy is relevant to address the research question?** | **Sample is representative of the target population?** | **Are the measurements appropriate?** | **Risk of nonresponse bias is low?** | **Statistical analysis appropriate to answer the research question?** | **Quality** | **Comments** |
| Descilo et al 2010 | No | Can't tell | Can't tell | Can't tell | Yes | Yes | Can't tell | Low | Overall unclear research question and rationale, although had acceptable response. |
| Evans et al 2018 | Yes | Yes | Yes | Can't tell | No | No | Yes | Low | No use of validated questionnaires (even in other populations) and adjustments to questionnaires noted without sufficient rationale. High risk of non-response bias. |
| Geltman et al 2005 | Yes | Yes | Yes | Can't tell | Yes | Can't tell | Yes | Medium | Limited discussion of nonresponse bias. |
| Grant-Knight et al 2009 | Can't tell | Can't tell | Can't tell | Can't tell | Yes | Can't tell | Yes | Low | Unclear research question, limited discussion of nonresponse bias. |
| Hasson III et al 2020 | Yes | Yes | Yes | Yes | Yes | Yes | Yes | High | High response rates, provided information regarding missing data. |
| Patel et al 2022 | Yes | Yes | Yes | Can't tell | Yes | No | Can't tell | Medium | Bias due to attrition. |
| Schapiro et al 2018 | Yes | Yes | Yes | Can't tell | Yes | No | Yes | Medium | Bias among missing datanon-response. |
| Vega Potler et al 2023 | Yes | Yes | Yes | Yes | Yes | Yes | Yes | High | Population discussed, research questions, clear, discussion of nonresponse. |

**TableS3.** Quality Assessment of Included Studies using Mixed-Method Assessment Tool
